# Immunohistochemical Expression of Mast Cell in Oral Reactive Lesions

**Published:** 2015-03

**Authors:** Setareh Shojaei, Shokoofeh Jamshidi, Ghodratollah Roshanaei, Shirin Modabbernia, Bahman Farzin

**Affiliations:** 1 Dept. of Oral and Maxillofacial Pathology, School of Dentistry, Hamadan University of Medical Sciences, Hamadan, Iran;; 2 Dental Research Center, Dept. of Oral and Maxillofacial Pathology, School of Dentistry, Hamadan University of Medical Sciences, Hamadan, Iran;; 3 Modeling of Noncommunicable Diseases Research Center, Dept. of Biostatistics and Epidemiology, School of Public Health, Hamadan University of Medical Sciences, Hamadan, Iran;; 4 Postgraduate Student, Dept. of Oral and Maxillofacial Pathology, School of Dentistry, Hamadan University of Medical Sciences, Hamadan, Iran;; 5 General Dentist, Private Practice, Hamadan, Iran;

**Keywords:** Mast Cell, Tryptase, Reactive, Lesion

## Abstract

**Statement of the Problem:**

Soft tissue reactive lesions are the most common lesions of the oral cavity. Although many studies have shown the interaction of mast cells with fibroblasts and their participation in fibrosis, the role of mast cells in these lesions is not well understood.

**Purpose:**

The aim of this study was to evaluate the mast cells (MCs) count in oral soft-tissue reactive lesions including peripheral giant cell granuloma (PGCG), peripheral ossifying fibroma (POF), irritation fibroma (IF) and normal oral mucosa.

**Materials and Method:**

In this cross-sectional study, 50 samples including IF, PGCG, POF (14 cases for each group) and 8 cases of normal oral mucosa were stained with tryptase antibody through immunohistochemistry. The number of mast cells was counted in 5HPF containing maximum counts for each section stained with tryptase. Statistical analysis including Chi-square test and Tukey test with a significance level of 0.05 were considered.

**Results:**

The number of MCs was found to have increased in reactive lesions compared with normal oral mucosa. MCs count in the POF group was higher than the others.

**Conclusion:**

These findings suggest a possible role of mast cells in the pathogenesis of reactive oral lesions and induction of fibrous tissues. Chemical mediators released from mast cells might influence other cells, especially fibroblasts, to induce fibrosis.

## Introduction

Soft tissue lesions of the oral cavity comprise a wide variety of lesions from reactive conditions to neoplasms. Reactive lesions of the oral soft tissues are the most common lesions that occur in the oral cavity and are derived from mesenchymal cells.[1] Considering their histopathological features, they may present fibrotic hyperplasia like in irritation fibroma or may show proliferation of granulation tissue like in pyogenic granulomas.[1] These lesions are developed in response to local irritations.[2] The most common reactive lesions of soft tissue are irritation fibroma, peripheral giant cell granuloma (PGCG), and peripheral ossifying fibroma (POF). Fibroma is the most prevalent tumor of the oral cavity.[2] There is still controversy whether fibroma is a real neoplasm or a reactive hyperplasia of the connective tissue in response to trauma and local irritations. Microscopic evaluation of irritation fibroma reveals nodular masses of collagenized and dense fibrous connective tissue.[2] PGCG is a relatively frequent tumor-like lesion of the oral cavity which is developed due to trauma or local irritation. Microscopic examination of PGCG exhibits a large number of multi-nucleated giant cells, in addition to proliferation of fibroblasts.[2] POF is a relatively common gingival hyperplasia that is composed of proliferation of fibroblasts along with mineralized components. These lesions are treated by conservative surgical excision.[2] Mast cells are components of the immune system, which originate from bone marrow and are found in all the connective and mucous tissues, especially around vessels, and in the peripheral and central nervous systems. They are round to oval in shape and are one-nucleated in histologic sections, with a large number of granules in the cytoplasm.[3-4] The primary role of these cells is to take part in hypersensitivity reactions and inflammatory processes. Evidence shows the role of mast cells in the formation of matrices, granulation tissue, wound healing and angiogenesis.[3-4] On the other hand, the study has shown interaction of mast cells with fibroblasts and their relation with the synthesis of collagen in a large number of pathologic lesions such as scleroderma, submucous fibrosis of the oral cavity, gingival fibromatosis and fibrotic changes in the salivary glands of patients with Sjögren’s syndrome.[3]

Several techniques are available to identify mast cells in different lesions, one of which is the use of immunohistochemical markers. In the present study, tryptase was used which is the most common serine protease of secretory granules of mast cells; it is an appropriate marker to identify these cells.[5-6] Since only a limited number of studies have been carried out on the role of mast cells in the pathogenesis of reactive lesions of soft tissues of the oral cavity[3, 7] and because reactive lesions are the most common lesions of oral cavity, the present study was undertaken to identify mast cell in such lesions, by using immunohistochemistry (IHC).

## Materials and Method

In this cross-sectional descriptive/analytical study, the archived files of Hamadan Faculty of Dentistry from 1996 to 2011 were evaluated. Fifty six samples of irritation fibroma (14 cases), peripheral giant cell granuloma (14 cases), peripheral ossifying fibroma (14 cases), and normal oral mucosa (14 cases) which had sufficient tissues and proper fixation were selected. Normal oral mucosa was selected from areas in vicinity of oral lesions that did not show hyperplasia or inflammation. Clinical data such as age, gender, and location of the lesions were recorded and analyzed. Slices with 4-µ in thickness were prepared from paraffin embedded sections for immunohistochemical staining. The slides were deparaffinized and rehydrated in Xylene and graded alcohol series. Endogenous peroxidase activity was blocked by incubating the slides in 1% H_2_O_2_ - methanol for 30 minutes.

Antigen retrieval was done in a microwave oven in citrate solution (0.01M, pH =6.0) for 35 minutes. Then, the microwave oven was turned off and the samples were retained in the same solution for 15 minutes. Ultimately, the slides were rinsed in distilled water for 5 minutes. The slides were incubated with monoclonal mouse anti-mast cell tryptase antibody (IgG1, Kappa; code: NCLMCTRYP-428, Novocastra, Newcastle upon Tyne, UK) diluted 1:150 for 20 min and pH= 6 at room temperature. Diaminobenzidine was used to produce brown staining followed by counterstaining with Mayer’s hematoxylin. For the negative control, the primary antibody was eliminated and replaced with PBC. Human tonsillar tissue was applied as positive control.

Two oral pathologists evaluated stained slides under a light microscope (Olympus BX41; Japan) at ×100 and ×400 magnifications. Five hot spot fields were selected under low magnification (×100) and mast cells were counted in each at ×400 magniﬁcation and the mean number of mast cells in the five regions was obtained.[8]

Data analysis was done using SPSS software (ver.16). The variance and Tukey test were used. Statistical significance was defined at *p*≤ 0.05.

## Results

Of all the samples evaluated, 22 belonged to men (44%) and 28 to women (56%). Out of all the PGCGs, 57.1% belonged to men; the samples of POFs and normal mucosa equally belonged to men and women (50%); and of all the irritation fibroma samples, 78.6% belonged to women. 

**Figure 1:**
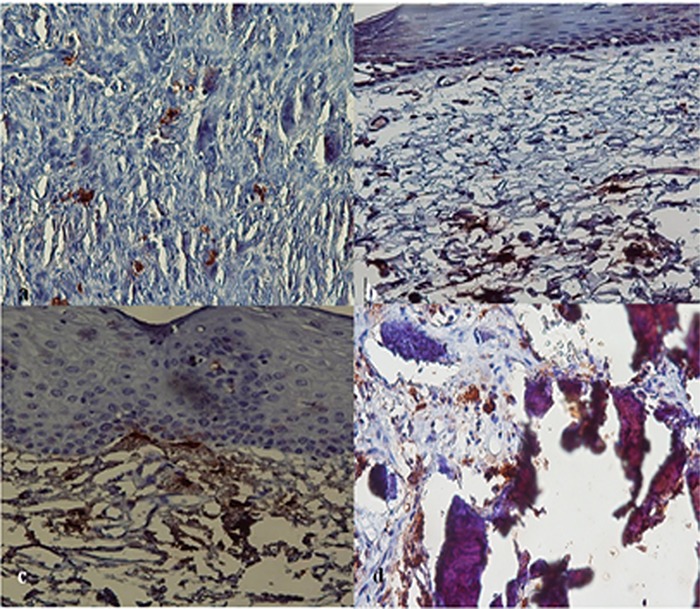
a: Immunoreactivity of the tryptase in peripheral giant cell granuloma (×400)  b: Immunoreactivity of the tryptase in reactive fibroma (×400)  c: Immunoreactivity of the tryptase in normal oral mucosa (×400)  d: Immunoreactivity of the tryptase in peripheral ossifying fibroma (×400)

The mean age of the subjects was 34±16.9 years, with a range of 7‒76 years. The most and least commonly involved areas were gingiva (48%) and the tongue (2%), respectively. The mean tryptase-positive mast cell count was 28.1±10.4 in POF, 23.5±7.9 in PGCG, 18.9±8.1 in irritation fibroma, and 13.7±4.2 in normal oral mucosa (Figure 1). Using variance analysis, statistically significant differences were obtained in mast cell counts between the study groups (*p*= 0.002, F=6), with the highest counts in POF (Table 1). Tukey tests showed statistically significant differences in mast cell counts among POF, irritation fibroma, and normal oral mucosa, (*p*= 0.005), (*p*= 0.002) respectively. However, there was no significant difference between POF and PGCG (*p*=0.15). In addition, there were no significant differences among irritation fibroma, normal oral mucosa (*p*= 0.17), and PGCG (*p*= 0.16).

**Table 1:** Means, standard deviations, and the amount of tryptase-positive mast cells in the study groups

**Lesion**	**Number**	**Mean**	**Standard** **deviation**	
Peripheral giant cell granuloma	14	23.5	7.9	*p*= 0.002
Peripheral ossifying fibroma	14	28.1	10.4	
Reactive fibroma	14	18.9	8.1	
Normal oral mucosa	8	13.7	4.2	
Total	50	22	9.5	

## Discussion

Mast cells have been identified in all organs and tissues of the human body and are considered as the sources of histamines, serine proteases (tryptase and kimase), and some other important chemical mediators.[5] Moreover, it has been shown that mast cells are responsible for the synthesis and secretion of histamine, heparin, and various proteases and chemical mediators such as prostaglandins, leukotrienes, and platelet-activating factors. Tryptase is the major protease of mast cells and has cytogenic effects on various cultured cells including smooth muscle cells and bronchial epithelial cells.[9] A study showed that tryptase induces COX2, resulting in proliferation of fibroblasts by Ca2+ and the non-CAMP-dependent mechanism to induce the activity of extracellular kinase isoforms (erkl/2).[10] In the present study, tryptase-positive mast cell counts in the lesions under study were more than those in the normal mucosa. Microscopic evaluations of reactive lesions of soft tissues showed an increase in collagen fibers.

Fibrosis, which is associated with an increase in the number of fibroblasts and myofibroblasts and in heavy deposits of extracellular matrix proteins, is the hallmark of a large number of oral lesions and pulmonary, hepatic, renal, cardiac, and skin disorders.[3, 11-13] Mast cells influence the function of fibroblasts and fibrosis is induced by the release of prefabricated mediators such as histamine, proteoglycans, metaproteolytic enzymes, newly formed mediators, and other fibrogenic cytokines such as platelet-derived growth factor (PDGF), tumor necrosis factor alpha (TNF-α), and basic fibroblast growth factor (b-FGF).[3]

Akers *et al.*[14] and Asano-Kato *et al.*[15] used the culture technique in their studies and showed that tryptase with protease activated receptor-2 resulted in proliferation of fibroblasts in the conjunctiva. In a study, Garbuzenko *et al.* revealed that human mast cells have a role in skin remodeling and fibrosis.[13]

In a rather similar study, Shahrabi *et al.* used toluidine blue staining of mast cells of fibroma, inflammatory fibrous hyperplasia, peripheral giant cell granuloma, peripheral ossifying fibroma, and normal gingival tissue and showed that the number of mast cells had increased in all groups compared with normal gingival tissue and their findings were similar to the results of the present study. However, despite the results of the current study, which showed statistically significant differences only between POF and normal mucosa, in that study there were significant differences between all the groups and normal gingiva regarding the number of mast cells.[3] The differences in these results might be attributed to the differences in the techniques used in these two studies to identify the mast cell. Metachromatic staining techniques -including toluidine blue- have lower specificity in identifying mast cells compared with IHC and might stain other cells such as macrophages and fibroblasts, as well. This technique is also unable to stain immature mast cells.[3]

Reddy *et al.* carried out a study on mast cells count in focal fibrous hyperplasia, peripheral giant cell granuloma, peripheral ossifying fibroma, pyogenic granuloma and normal gingival tissue by using toluidine blue staining. They reported that the number of mast cells increased in all groups compared with normal gingival tissue and mast cells count was more in POF and fibrous hyperplasia. They also suggested that mast cells may lead to fibrosis in oral reactive lesions.[7] Despite the results of the present study, there were significant differences between all the groups and normal gingiva in their study. Similar to the Shahabi *et al.’s* study, Reddy *et al.* used the histochemical method in their study.[3, 7] As it was mentioned, immunohistochemical staining method is more specific than the histochemical method in mast cells count.

In a study by Santos *et al.* on giant cell fibroma (GCF), inflammatory fibrous hyperplasia (IFH), and normal oral mucosa, mast cells were identified in all samples despite the differences in the type of reactive lesions between that study and the present one.[5] In a study by Epivatianos *et al.* on chronic sialoadenitis of the submandibular gland, more tryptase-positive mast cells were observed in submandibular gland with chronic sialoadenitis compared with normal submandibular gland, especially in glands with more fibrous sialoadenitis, and the difference was statistically significant.[11] The results of that study were consistent with those of the current one considering the increase in mast cell counts in fibrous tissues compared with normal samples.

In the studies by Andersson *et al.*[12] and Sabarinath *et al*.[16] on the fibrous lesions of the oral mucosa and lungs, the number of mast cells was higher in fibrous lesions similar to the results of the present study. Some studies have shown the interaction of mast cells and fibrosis in adipose tissue and kidney.[17-18] The results of our study and majority of other mentioned studies indicate an increase in mast cell counts in fibrous tissues. In the present study, the number of mast cells in POF was higher than that in PGCG, and the difference was statistically significant.[7, 12, 16] Since the fibrous tissue in POF is higher than PGCG, it may show a possible role of mast cells in fibrosis, though mast cell counts were lower in fibroma compared with POF and PGCG.

Even though mast cells are important factors in the induction of fibrosis, it seems that fibrosis occurs due to the interaction of mast cells with other cells, especially with fibroblasts. 

## Conclusion

Based on the presence of mast cells in all the samples under study and the increase in mast cell counts in reactive lesions of the oral cavity compared with normal oral mucosa, it can be concluded that mast cells might have a role in the pathogenesis of reactive oral lesions and induction of fibrous tissues. On the other hand, chemical mediators released from mast cells might influence other cells, especially fibroblasts to induce fibrosis. 
